# Clinicopathological features and prognosis of young gastric cancer patients following radical gastrectomy: a propensity score matching analysis

**DOI:** 10.1038/s41598-019-42406-4

**Published:** 2019-04-11

**Authors:** Wu Liu, Hu Quan, Xiaoyan Chen, Yongzhong Ouyang, Hua Xiao

**Affiliations:** 1 Department of Gastroenterology and Urology, Hunan Cancer Hospital and the Affiliated Cancer Hospital of Xiangya School of Medicine, Central South University, Changsha, China; 2 Department of Gastroduodenal and Pancreatic Surgery, Hunan Cancer Hospital and the Affiliated Cancer Hospital of Xiangya School of Medicine, Central South University, Changsha, China; 3 Department of Pathology, Hunan Cancer Hospital and the Affiliated Cancer Hospital of Xiangya School of Medicine, Central South University, Changsha, China

## Abstract

The aim of the this retrospective study was to investigate the clinicopathological features of gastric cancer (GC) in young patients and the potential impact of age on the prognosis of patients undergoing radical gastrectomy for GC. From November 2010 to November 2016, 317 young (≤45 years) and 1344 older patients (>45 years) who underwent radical gastrectomy for stage I-III GC were enrolled. The association between age and prognosis was estimated by univariate and multivariate analyses after propensity score matching (PSM). Compared with older patients, the proportion of females, poorly differentiated tumors, good nutritional status, and who received neo-adjuvant and/or adjuvant chemotherapy was significant higher in younger patients, but were less likely to suffer from comorbidities or post-operative complications (all *P* < 0.05). PSM analysis created 310 pairs of patients. After matching, the long-term survival in younger patients was significantly longer than in older patients at stage I, but similar at stages II and III. However, a young age was not identified as a significant prognostic factor. In conclusion, the prognosis of young GC patients is comparable with and even better than that in older patients after radical gastrectomy when matched for baseline characteristics. Early detection could improve the prognosis of young GC patients.

## Introduction

Gastric cancer (GC) is the fourth most frequently diagnosed malignancy worldwide and is ranked as the second leading cause of cancer-related death in China^1,2^. Despite a steady decrease in the incidence of GC over recent decades, the incidence and mortality of GC among young patients is increasing in both Eastern and Western countries^3–5^. Previous studies have revealed that 5.2–19.8% of GC occurred in patients aged ≤45 years^6–11^. Although the unique clinicopathological features of GC in young patients have been defined in a number of studies, including female predominance, diffuse histological types, lymph node metastasis, poorer differentiation and unresectability^6–11^, the prognosis of young GC patients after gastrectomy remains controversial. A number of authors have argued that young GC patients have a poorer prognosis mainly because of more aggressive tumor behaviors and delayed diagnosis^9,10,12–14^. However, other researchers found that age was not an independent predictor for survival, and the oncological outcomes of young GC patients was similar or even better than those of older patients^6,7,11,15–17^. Possible explanations for the conflicting data were the variable definitions of young age and inconsistency in patient inclusion criteria. We hypothesize that the poorer prognosis for young GC patients are not necessarily due to age itself, but may be related to other related prognostic factors, such as an advanced tumor stage and more aggressive tumor behaviors. The aim of this retrospective study was to investigate the clinicopathological features of GC in young patients and the potential influence of age on overall survival (OS) and disease-free survival (DFS) in patients who underwent radical gastrectomy for GC, based on a prospectively registered high-volume sample database in China. Multivariate Cox regression and propensity score matching (PSM) analyses were used to investigate the potential influence of age on prognosis.

## Methods

### Design and patients

**Figure 1:**
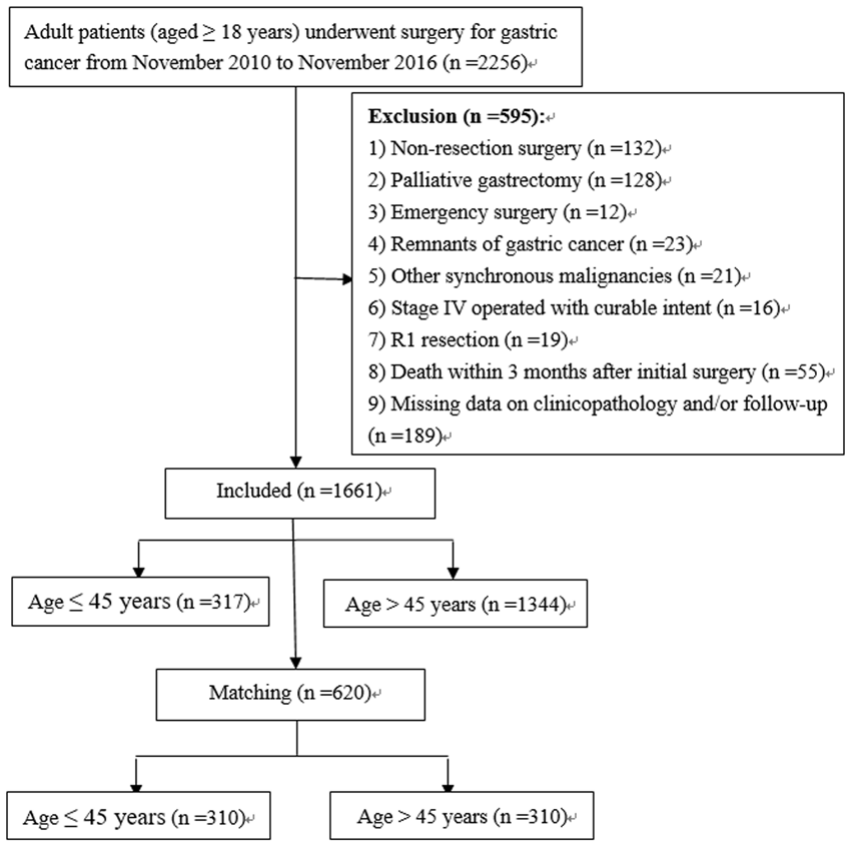
Flow-chart.

The medical records of 2256 patients ≥18 years of age undergoing operations for pathologically identified gastric adenocarcinoma in the Hunan Cancer Hospital between November 2010 and November 2016 were reviewed. Figure 1 shows the exclusion criteria and flow-chart of the present study. The ethics committee of Hunan Cancer Hospital approved this study and waived the need for informed consent considering the retrospective and observational nature of the study design.

### Peri-operative management and follow-ups

Gastrointestinal surgeons with sufficient experience of gastrectomy and D2 lymphadenectomy performed or supervised all of the operations, in accordance with the Japanese Gastric Cancer treatment guidelines^18^. The main peri-operative management and follow-ups have been described in our previous studies^19,20^. Briefly, for patients with early GC, gastrectomy with D1 or D1 + lymph node dissection was performed by laparoscopy or a laparoscopy-assisted procedure. While for most patients with advanced GC, a midline incision and laparotomy with D2 or D2 + lymphadenectomy was the primary surgical type. Combined multi-organ resection was considered to achieve R0 resection when deemed necessary. Neo-adjuvant chemotherapy was applied in 91 patients with stage cT3–4N + GC, in a standard manner with S-1 plus oxaliplatin (SOX), or epirubicin, cisplatin plus fluorouracil (ECF) as the main regimens for 2 to 4 cycles before surgery^18,21^. For those patients with post-operatiive pathologically diagnosed stage II/III or T1N1M0, adjuvant chemotherapy (fluorouracil and platinum-based regimens) was administered within 6 months of their operations^18^. Patients were followed-up every 3-months for 1 to 2 years, and every half-year for 3 to 5 years, and then annually thereafter until December 2017. Physical examination and serum tumor markers were measured at each follow-up. Computed tomography (CT) scans and/or ultrasonography were carried out at 6-month intervals during the 5 years after surgery and endoscopy was performed at 2-year intervals. Magnetic resonance imaging (MRI), positron emission tomography and/or biopsy was performed when distant metastasis was suspected. Chemotherapy, radiochemotherapy, molecular targeted drugs, traditional Chinese herbal drugs, and conservative treatment, either alone or in combination, were the main treatments for those with tumor recurrence. Very few patients had the opportunity to undergo re-section.

### Definitions and data collection

Young patients were defined as individuals who ≤45 years old at the time of surgery, as reported by previous studies^6–10^. Staging was performed in conformity with the 7th Union for International Cancer Control (UICC) tumor, node, metastasis (TNM) classification^22^. Complications were defined as morbidity and mortality within 30 days after surgery and defined according to Clavien-Dindo classification^23^. Major complications were defined as Clavien-Dindo grade III or greater. For those patients developed multiple complications, Clavien-Dindo stage was classified according to the most severe one. OS was calculated from the time of gastrectomy until death or the last follow-up. DFS was measured from the time of gastrectomy to the date at which the first recurrence was identified. Clinicopathological characteristics including patients’ demographics, operative and oncologic details, and survival data were obtained from our prospectively registered database.

### PSM

To balance any differences in baseline characteristics between the 2 groups, young patients were matched to old patients using the PSM method by a logistic regression model, as previously described^24,25^. The propensity score for each patient was calculated, taking into account that the basic variables differed significantly between younger and older patients, such as gender, the American Society of Anesthesiologist (ASA) score, any comorbidities, neo-adjuvant chemotherapy, preoperative albumin levels, differentiation stage, postoperative complications and adjuvant chemotherapy. Nearest neighbor matching was performed in a 1:1 ratio without replacement and a caliper width with a 0.01 standard deviation (SD) was specified.

### Statistical analysis

Data were analyzed using SPSS software (Ver. 24.0, IBM Corporation, New York, US). Baseline categorical and continuous variables are presented as means ± SD and numbers (%), and were compared using a *t*-test, Fisher’s exact or χ^2^ tests, as appropriate. OS and DFS rates were estimated by the Kaplan-Meier method. The differences between survival curves were evaluated by a long-rank test. Associations between variables and OS or DFS were assessed by univariate and multivariate Cox proportional hazard regression analysis. Variables that were considered to be potentially important for univariate analysis (*P* ≤ 0.05) and age were entered into the multivariate analyses. A result was considered to be statistically significant when *P* < 0.05.

### Ethical approval

All procedures performed in studies involving human participants were in accordance with the ethical standards of the institutional and/or national research committee and with the 1964 Helsinki declaration and its later amendments or comparable ethical standards.

## Results

### Baseline characteristics

As shown in Figure 1, a total of 1661 GC patients at stage I-III who underwent radical gastrectomy were analyzed. Of these, 317 cases (19.1%) were ≤45 years of age (young group) at the time of surgery, and the remaining 1344 cases (80.9%) were >45 years (old group). The clinicopathological characteristics of the 1661 patients are shown in Table 1. Compared to old patients, young GC patients had a slight female predominance, with less ASA scores, lower proportions of comorbidities and post-operative complications, more likelihood of receiving neo-adjuvant and/or adjuvant chemotherapy, with higher pre-operative albumin levels, but poorer histological classification of their tumors (all *P* < 0.05).

**Table 1:** Clinicopathological characteristics of the entire study cohort stratified by age greater than 45 years or not, before and after propensity score matching.

Variables	Total cohort (n = 1661)			Propensity score matched cohort (n = 620)		
	Age ≤ 45 years (n = 317)	Age > 45 years (n = 1344)	*P* value	Age ≤ 45 years (n = 310)	Age > 45 years (n = 310)	*P* value
Gender (males)	156 (49.2%)	939 (69.9%)	<0.001	153 (49.4%)	133 (42.9%)	0.107
Age (years)	39.49 ± 5.43	58.78 ± 7.77	<0.001	39.60 ± 5.34	56.38 ± 6.82	<0.001
Body Mass Index (kg/m^2^)	21.77 ± 3.00	21.77 ± 2.93	0.992	21.80 ± 2.97	21.95 ± 2.91	0.528
ASA score			<0.001			0.551
1	82 (25.8%)	125 (9.3%)		78 (25.2%)	67 (21.6%)	
2	232 (73.2%)	1018 (75.7%)		229 (73.9%)	241 (77.7%)	
3	3 (0.9%)	195 (14.5%)		3 (1.0%)	2 (0.6%)	
4	0 (0.0%)	6 (0.4%)		0 (0.0%)	0 (0.0%)	
Any comorbidities	65 (20.5%)	428 (31.8%)	<0.001	65 (21.0%)	76 (24.5%)	0.292
Neo-adjuvant chemotherapy	31 (9.8%)	60 (4.5%)	<0.001	26 (8.4%)	33 (10.6%)	0.338
Complication due to the tumor*	64 (20.2%)	305 (22.7%)	0.335	62 (20.0%)	54 (17.4%)	0.410
Pre-operative hemoglobin (g/L)	118.94 ± 25.10	119.27 ± 24.06	0.824	118.85 ± 25.19	117.45 ± 22.44	0.465
Pre-operative albumin (g/L)	39.10 ± 4.46	37.79 ± 4.60	<0.001	39.08 ± 4.44	38.69 ± 4.32	0.271
Type of resection			0.495			0.151
Subtotal gastrectomy	250 (78.9%)	1036 (77.1%)		247 (79.7%)	232 (74.8%)	
Total gastrectomy	67 (21.1%)	308 (22.9%)		63 (20.3%)	78 (25.2%)	
Laproscopy surgery	44 (13.9%)	154 (11.5%)	0.231	42 (13.5%)	37 (11.9%)	0.547
Intra-operative blood loss (mL)	192 ± 107	206 ± 120	0.056	192 ± 108	207 ± 97	0.073
Operation time (min)	203.22 ± 57.75	201.49 ± 53.09	0.609	203.30 ± 58.19	200.26 ± 52.67	0.496
Tumor size (cm)	4.02 ± 2.30	4.09 ± 2.06	0.613	4.01 ± 2.31	4.11 ± 1.91	0.547
Lymph node harvested	21.88 ± 8.30	21.31 ± 8.71	0.292	21.95 ± 8.36	21.79 ± 8.97	0.820
Tumor location			0.070			0.226
Upper	25 (7.9%)	111 (8.3%)		24 (7.7%)	38 (12.3%)	
Middle	82 (25.9%)	258 (19.2%)		78 (25.2%)	81 (26.1%)	
Lower	199 (62.8%)	922 (68.6%)		197 (63.5%)	178 (57.4%)	
Diffuse	11 (3.5%)	53 (3.9%)		11 (3.5%)	13 (4.2%)	
Differentiation			0.001			0.100
Well-differentiated	36 (11.4%)	263 (19.6%)		35 (11.3%)	49 (15.8%)	
Moderate- or poor-differentiated	281 (88.6%)	1081 (80.4%)		275 (88.7%)	261 (84.2%)	
T stage^†^			0.099			0.097
T1	69 (21.8%)	265 (19.7%)		67 (21.6%)	49 (15.8%)	
T2	33 (10.4%)	214 (15.9%)		33 (10.6%)	50 (16.1%)	
T3	15 (4.7%)	63 (4.7%)		15 (4.8%)	14 (4.5%)	
T4	200 (63.1%)	802 (59.7%)		195 (62.9%)	197 (63.5%)	
N stage^†^			0.322			0.096
N0	123 (38.8%)	547 (40.7%)		122 (39.4%)	107 (34.5%)	
N1	53 (16.7%)	228 (17.0%)		51 (16.5%)	53 (17.1%)	
N2	53 (16.7%)	259 (19.3%)		51 (16.5%)	75 (24.2%)	
N3	88 (27.8%)	310 (23.1%)		86 (27.7%)	75 (24.2%)	
pTNM stage^†^			0.467			0.306
I	79 (24.9%)	369 (27.5%)		78 (25.2%)	62 (20.0%)	
II	71 (22.4%)	265(19.7%)		69 (22.2%)	73 (23.5%)	
III	167 (52.7%)	710 (52.8%)		163 (52.6%)	175 (56.5%)	
Perioperative blood transfusion	56 (17.7%)	269 (20.0%)	0.343	55 (17.7%)	57 (18.4%)	0.835
Post-operative complications^‡^	15 (4.7%)	127 (9.4%)	0.007	15 (4.8%)	23 (7.4%)	0.180
Minor (Grade II)	10 (3.2%)	95 (7.1%)		10 (3.2%)	16 (5.2%)	
Major (Grade III or greater)	5 (1.6%)	32 (2.4%)		5 (1.6%)	7 (2.3%)	
Adjuvant chemotherapy	237 (74.8%)	764 (56.8%)	<0.001	230 (74.2%)	243 (78.4%)	0.220

Data are presented as mean ± SD or n (%). ASA, American Society of Anesthesiologist. *Including pyloric obstruction or bleeding.

^†^Tumor stages are based on 7th edition of the Union for International Cancer Control TNM classification.

^‡^Based on the Clavien-Dindo severity classification of surgical complications.

Among the 317 young patients in the present study, the median age was 40 years (range, 19–45), 27 cases were ≤30 years of age and 165 cases ≤40 years old. Slightly more of the cohort of patients was female (50.8%). Thirty-one patients (9.8%) underwent neo-adjuvant chemotherapy (fluorouracil + platinum). Concerning the histological classifications, moderate to poorly differentiated tumors (281 cases, 88.6%) were clearly predominant. The majority of patients were diagnosed at an advanced tumor stage, including 200 cases (63.1%) with serosal invasion, 194 cases (61.2%) with lymph node metastasis and 167 cases (52.7%) of pathological TNM stage III. After surgery, most of the patients (214 cases, 89.9%) with stage II/III GC received fluorouracil and platinum based adjuvant chemotherapy within 6 months of surgery.

There was no significant association between young age (≤45 years) and OS or DFS among the entire 1661 patients by long-rank test (*P* = 0.724 and 0.661, respectively). After adjusting for potential confounding factors (including variables with a *P* value ≤ 0.05 in univariate analysis) by a multivariate Cox regression analysis, young age was not identified to be an independent risk factor (*P* = 0.882 and 0.483, respectively) (Supplementary Tables 1 and 2).

### PSM analysis

After a 1:1 matching according to the propensity score, 310 young patients were matched to 310 patients in the old group. The basic covariates between the 2 groups in the matched data are listed in Table 1. After matching, all of the baseline characteristics became comparable between the 2 groups (*P* > 0.05), except for mean age (39.60 ± 5.34 years *vs* 56.38 ± 6.82 years, *P* < 0.001), which was not entered into the subsequent PSM analysis.

### Survival analysis

**Figure 2:**
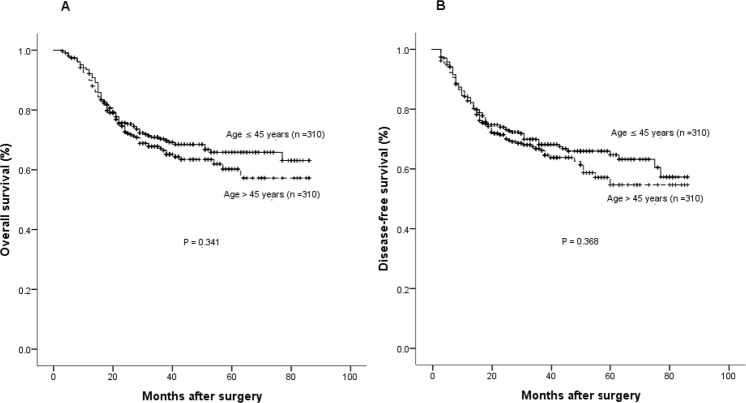
Survival cures of the young (age ≤ 45 years) and old (age > 45 years) groups in the propensity score matched cohort. (**A**) Overall survival (*P* = 0.341 by log-rank test). (**B**) Disease-free survival (*P* = 0.368 by log-rank test).

After median observation periods of 27 months (range, 3–86), 190 cases (30.6%) in the matched 620 patients died, including 176 deaths due to GC (92.6%), with 91 (29.3%) deaths in the young group and 99 (31.9%) in the old group (*P* = 0.486), respectively. OS rates in young patients at 1, 3, and 5 years were 93.0%, 70.3%, and 65.3% respectively, which were comparable to those in the old group (92.1%, 67.2% and 58.7%, *P* = 0.341) (Figure 2A). As shown in Table 2, there was no significant association between young age (≤45 years) and OS among the matched 620 patients after univariate analysis (*P* = 0.341). After adjusting for potential confounding factors (including variables with a *P* value ≤ 0.05 in univariate analysis) by a multivariate Cox regression analysis, young age was not identified to be a significant predictive factor (*P* = 0.893).

**Table 2:** Univariate and multivariate analyses of prognostic factors for overall survival after radical resection of gastric cancer in the propensity matched cohort (n = 620).

Variables	N	3-,5-year OS rates	UV *P* value	MV HR (95% CI)	MV *P* value
Gender			0.970		
Male	286	69.2%, 63.8%			
Female	334	68.7%, 62.3%			
Age (years)			0.341		0.893
>45	310	67.2%, 58.7%			
≤45	310	70.3%, 65.3%			
Body mass index (kg/m^2^)			0.010		0.061
≥25	89	80.2%, 66.3%			
<25	531	66.6%, 60.2%			
Any comorbidities			0.791		
Yes	141	71.2%, 64.6%			
No	479	67.8%, 61.8%			
Pre-operative hemoglobin (g/L)			0.011		0.866
≥100	489	70.9%, 66.1%			
<100	131	58.6%, 45.1%			
Pre-operative albumin (g/L)			0.403		
≥35	527	68.4%, 64.7%			
<35	93	69.5%, 54.2%			
Neo-adjuvant chemotherapy			0.105		
Yes	59	64.4%, 53.6%			
No	561	70.7%, 64.8%			
Surgical procedure			0.079		
Laproscopy	79	80.6%, 80.6%			
Open	541	67.6%, 61.5%			
Type of resection			<0.001		0.541
Total gastrectomy	141	46.9%, 42.4%			
Sub-total gastrectomy	479	75.3%, 69.2%			
Operation time (min)			<0.001		
≥240	133	54.4%, 50.4%		1.439 (1.046–1.979)	0.025
<240	487	72.7%, 65.8%			
Intra-operative blood loss (mL)			0.005		0.394
≥300	121	57.0%, 53.9%			
<300	499	71.7%, 64.6%			
Tumor location			<0.001		<0.001
Lower third	368	79.1%, 71.9%		1.894 (1.397–2.567)	
Upper, middle third or diffused	252	53.4%, 47.2%			
Differentiation			0.009		0.061
Well-differentiated	84	80.5%, 75.4%			
Moderate- or poor-differentiated	536	66.9%, 60.7%			
Tumor size (cm)			<0.001		0.282
≥5	226	52.8%, 44.6%			
<5	394	78.1%, 72.4%			
Depth of invasion			<0.001		<0.001
T4	392	56.8%, 49.8%		3.198 (2.023–5.055)	
T1-3	228	90.2%, 86.1%			
Lymph node metastasis			<0.001		<0.001
Yes	391	56.2%, 49.6%		3.424 (2.208–5.307)	
No	229	90.6%, 85.7%			
Peri-operative blood transfusion			<0.001		0.046
Yes	112	52.2%, 45.3%		1.394 (1.005–1.933)	
No	508	72.0%, 65.3%			

OS, overall survival; CI, confidence interval; HR, hazard ratio; UV, univariate analysis; MV, multivariate analysis.

Tumor recurrence occurred in 201 patients (32.4%) during the observation period, with 97 cases (31.3%) in the young group and 104 cases (33.5%) in the old group (*P* = 0.548). The DFS rates at 1-, 3- and 5-years in the young patients were 83.9%, 68.2%, 64.7%, and clearly similar to those in old patients (84.8%, 66.3%, 54.8%, respectively, *P* = 0.368) (Figure 2B). Regarding OS, young age was not significantly related to DFS, confirmed by univariate and multivariate Cox regression analysis (*P* = 0.969) (Table 3).

**Table 3:** Univariate and multivariate analyses of prognostic factors for disease-free survival after radical resection of gastric cancer in the propensity matched cohort (n = 620.

Variables	N	3-, 5-year DFS rates	UV *P* value	MV HR (95% CI)	MV *P* value
Gender			0.407		
Male	286	68.5%, 63.0%			
Female	334	67.1%, 58.0%			
Age (years)			0.368		0.969
>45	310	66.3%, 54.8%			
≤45	310	68.2%, 64.7%			
Body mass index (kg/m^2^)			0.013		0.078
≥25	89	76.4%, 62.1%			
<25	531	65.7%, 58.0%			
Comorbidities			0.392		
Yes	141	66.3%, 63.3%			
No	479	67.2%, 59.5%			
Pre-operative hemoglobin (g/L)			0.007		0.138
≥100	489	70.3%, 64.2%			
<100	131	55.2%, 44.5%			
Pre-operative albumin (g/L)			0.362		
≥35	527	68.2%, 62.3%			
<35	93	64.3%, 53.4%			
Neo-adjuvant chemotherapy			0.101		
Yes	59	62.1%, 51.2%			
No	561	68.7%, 62.0%			
Surgical procedure			0.031		0.670
Laproscopy	79	76.5%, 76.5%			
Open	541	66.0%, 58.5%			
Type of resection			<0.001		0.344
Total gastrectomy	141	43.3%, 39.2%			
Sub-total gastrectomy	479	74.4%, 66.8%			
Operation time (min)			<0.001		
≥240	133	53.4%, 46.8%		1.486 (1.089–2.028)	0.012
<240	487	70.9%, 64.2%			
Intra-operative blood loss (mL)			0.021		0.524
≥300	121	56.1%, 52.0%			
<300	499	69.9%, 62.0%			
Tumor location			<0.001		<0.001
Lower third	368	78.3%, 70.5%		1.8940 (1.411–2.533)	
Upper, middle third or diffused	252	51.4%, 45.2%			
Differentiation			0.019		0.152
Well-differentiated	84	77.8%, 70.8%			
Moderate- or poor-differentiated	536	65.8%, 58.5%			
Tumor size (cm)			<0.001		0.127
≥5	226	50.3%, 42.6%			
<5	394	77.0%, 71.5%			
Depth of invasion			<0.001		<0.001
T4	392	55.0%, 46.6%		3.297 (2.127–5.112)	
T1-3	228	89.4%, 85.0%			
Lymph node metastasis			<0.001		<0.001
Yes	391	54.6%, 48.0%		3.878 (2.506–6.001)	
No	229	89.6%, 82.5%			
Peri-operative blood transfusion			<0.001		0.078
Yes	112	51.5%, 43.4%			
No	508	70.9%, 64.3%			

DFS, disease-free survival; CI, confidence interval; HR, hazard ratio; UV, univariate analysis; MV, multivariate analysis.

### Subgroup analysis of survival according to the tumor stage

**Figure 3:**
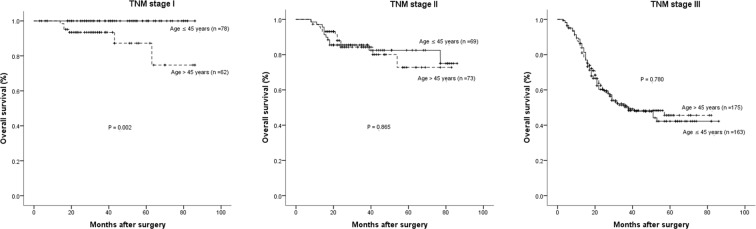
Overall survival cures of the young (age ≤ 45 years) and old (age > 45 years) groups according to each tumor stage in the propensity score matched cohort. Stage I (*P* = 0.002 by log-rank test), Stage II (*P* = 0.865 by log-rank test) and Stage III (*P* = 0.780 by log-rank test).

**Figure 4:**
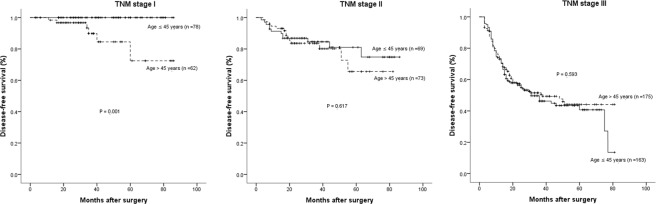
Disease-free survival cures of the young (age ≤ 45 years) and old (age > 45 years) groups according to each tumor stage in the propensity score matched cohort. Stage I (*P* = 0.001 by log-rank test), Stage II (*P* = 0.617 by log-rank test) and Stage III (*P* = 0.593 by log-rank test).

The distribution of pathological stages of the matched 620 patients was as follows: stage IA, 88 patients (14.2%); stage IB, 53 patients (8.5%); stage IIA, 38 patients (6.1%); stage IIB, 105 patients (16.9%); stage IIIA, 79 patients (12.7%); stage IIIB, 103 patients (16.6%); and stage IIIC, 154 patients (24.8%). Interestingly, among patients with stage I GC, the 5-year OS and DFS rates were 100% and 100% in young patients, which were significant greater than in old patients (76.8% and 72.5%, *P* = 0.002 and *P* = 0.001, respectively). With regard to stages II and III, the 5-year OS and DFS rates were all comparable between young and old patients. Figures 3 and 4 show the OS and DFS curves at each stage.

## Discussion

Although a number of studies have investigated the clinicopathological characteristics of GC in young patients and the impact of age on the oncological outcomes, the conclusions are contradictory and perhaps even confusing^9–17^. Wang *et al*.^16^ conducted a retrospective analysis of 3930 GC patients who underwent radical gastrectomy including 342 cases (8.7%) aged ≤40 years at the time of surgery, and concluded that the 5-year OS in young patients was longer than in old patients, despite young patients exhibiting more aggressive tumors and a higher recurrence rate. Another retrospective study of 1124 patients reported that both the OS and DFS rates were comparable between the patients aged 40 years or younger (n = 115) and the remaining 1009 patients aged between 56 and 75 years^15^. But there were also several studies that revealed prognosis was poorer in young GC patients than in older ones, mainly due to more aggressive tumor behaviors and the advanced stage of the disease^9,10,12–14^. Possible explanations for the conflicting results were the variable definitions of young age and the inconsistency in patient inclusion criteria. Young age was defined as younger than 30, 40, 45, or 50 years old in previous studies^3,6–11,13,15–17,26^. Considering the extremely low incidence of GC in patients under 40 years and the mean age of patients in the entire cohort of the present study was 55.1 years, we selected 45 years as the cut-off age to define young patients, as reported by the majority of previous studies^6–10^.

The potential impact of age on prognosis may be difficult to clarify in patients with stage IV GC, who experienced widespread metastases and extremely disappointing survival times, but were included in most of the studies^6–9,15,16^. In addition, the majority of the previously published literature that investigated the relationship between age and GC prognosis was based on small sample sizes, with a long study time period over 10 years, and lacking of recent data, which may affect the accuracy and precision of the Cox regression model and the reliability of conclusions drawn^6,9–11^. To the best of our knowledge, our study is the first conducted to investigate the potential impact of age on prognosis based on patients who were managed after 2010, with large sample size, and using PSM analysis. Such a study is necessary because advances in surgical techniques (such as laparoscopy procedures), establishment of D2 lymph node dissections, neo-adjuvant chemotherapy and adjuvant chemotherapies for advanced GC occurred in the past decade.

Consistent with previous studies, the clinicopathological features between young and old GC patients varied significantly before matching. Some of these factors, such as histologic classification of the tumor, pre-operative immunological and nutritional status, post-operative complications, and especially adjuvant chemotherapy, have been confirmed to play an essential role in prognosis after gastrectomy for GC^27–29^. Thus, the association between age and survival may be confused by these factors. Therefore, we explored the potential influence of young age on survival of patients who underwent radical gastrectomy, using PSM and multivariate Cox regression analyses to make the clinicopathological characteristics between young and old patients comparable, and to investigate the impact of other putative risk factors.

PSM has been proposed as a statistical technique to deal with confounding bias and to mimic a randomized clinical trial, and as a result increase the evidence level in observational studies^24,30^. As shown in Table 1, the differences of the basic factors between young and old patients were balanced out after matching. Young age was identified not to be a significant predictor for decreased OS or DFS by further multivariate Cox regression analyses (*P* = 0.893 and 0.969, respectively) among the matched 620 patients. In contrast, the tumor stage and operation-related factors such as lymph node metastasis, serosal invasion, longer duration of surgery and peri-operative transfusion were identified to be more important risk factors for long term survival than age in GC patients after radical gastrectomy. Thus, the negative influence of young age on prognosis of GC patients defined by previous studies is likely not associated with age itself but rather with the tumor behavior and operation-associated factors. PSM in combination with multivariate Cox regression analyses can offer more powerful statistical strength and thus make the final conclusions more robust and reliable^25^.

The proportion of young GC patients in the present study was 19.1%, which was comparable with the 19.5% and 19.8% reported by Park *et al*. and Braga-Neto *et al*.^7,10^, but significantly greater than the 5.2% and 8.5% reported in other studies^9,12^. One possible explanation is the relatively high incidence of GC in Eastern Asia, and the mean age at diagnosis is significant younger in patients than in Western countries^20,31^. In general, GC more commonly occurred in men in old patients whereas in young patients, female are affected at the same rate or more often than males. Although the reason for the increased frequency in females is still unknown, scholars have argued that this might be hormonally related^32^. As reported in previous studies, GC in young patients presents with more aggressive histological characteristics, such as poor differentiation, but this finding was not confirmed to be significantly associated with OS or DFS in the present study.

Although a few studies have investigated the prognosis of young GC patients, some of them have taken all stages of the tumor in to account. In fact, long-term survival between each stage could be significantly different, especially in those patients with distant metastasis who received unresection treatments. Thus we compared the survival rates at 1-, 3- and 5-years between young and old patients according to the tumor stage. Interestingly, both of the 5-year OS and DFS rates were 100% in young GC patients at stage I, which were significant higher than those in the older patient group. The reason is probably because young patients have better immunological and nutritional statuses, which may play an essential role in the prognosis of GC, independent of age, the TNM stage and histology^27^. Whereas for patients at stage II/III, obviously more young patients received neo-adjuvant and/or postoperative chemotherapy compared with older patients before matching (9.8% *vs* 4.5%, *P* < 0.001; 89.5% *vs* 72.1%, *P* < 0.001), which may significantly affect the prognosis. Moreover, postoperative complications, such as intra-abdominal infection, which have been identified to have negative impacts on the prognosis of GC patients after radical gastrectomy^28^, were significantly more common in old patients. Whereas after matching, similar percentages of patients who received neoadjuvant (8.4% *vs* 10.6%, *P* = 0.338) and/or adjuvant chemotherapy (91.8% *vs* 89.9%, *P* = 0.803) had comparable postoperative complications (4.8% *vs* 7.4%, *P* = 0.180) in the 2 groups. Further analysis revealed that prognosis was comparable for stage II and III GC between young and old patients (Figures 3 and 4). Although moderate- or poor-differentiated tumor was more common to see in young patients as shown in Table 1, the lower incidence of postoperative complications and higher rates of receiving adjuvant chemotherapy might partly explain the similar prognosis between the 2 groups, a conclusion echoed by Hsieh *et al*.^15^.

The limitations of the present study should be noted. First, the major limitation was its retrospective nature and single-institution design. Second, some important variables which may affect the prognosis of GC patients were not included in this study, such as the Lauren classification, the exact treatments (such as chemotherapy, radiochemotherapy, and reoperation) after tumor recurrence, and molecular profile of these patients, thus the possible reasons lie behind the association between age and prognosis of GC could not be clarified, and further studies are needed. Third, the median follow-up time (27 months) was relatively short, especially for stage I GC patients, in whom the 5-year survival rate was reported to be over 95% in some centers^33^. Fourth, only patients without distant metastasis and undergoing radical gastrectomy were included. Previous studies have reported that stage IV and unresectable disease was significantly more common in young GC patients at diagnosis^7,10^. Thus, our conclusions must be interpreted with caution when applied to patients with stage IV disease. Last but by no means least, although PSM analysis has the advantage of balancing the baseline characteristics between the 2 groups, it restricts the analysis to a relatively small sample size, thus inevitably limiting the statistical power.

## Conclusions

The prognosis is comparable with, and even better than, that of old patients after radical gastrectomy, according to the tumor stage, identified by a combination of PSM. and multivariate Cox regression analyses. Early detection should improve the long-term survival of young patients with GC.

## Supplementary information

Supplementary Table 1 and Table 2

## References

[1] Torre LA (2015). Global cancer statistics, 2012. CA Cancer J Clin.

[2] Chen W (2016). Cancer statistics in China, 2015. CA Cancer J Clin.

[3] Strong VE (2017). Comparison of young patients with gastric cancer in the United States and China. Ann Surg Oncol.

[4] Yin. J (2017). Gastric cancer mortality trends in China (2006–2013) reveal increasing mortality in young subjects. Anticancer Res.

[5] Islami, F., DeSantis, C. E. & Jemal, A. Incidence trends of esophageal and gastric cancer subtype by race, ethnicity, and age in the United States, 1997–2014. *Clin Gastroenterol Hepatol*, 10.1016/j.cgh.2018.05.044 Jun 11 2018. 10.1016/j.cgh.2018.05.044 29902641

[6] Santoro R, Carboni F, Lepiane P, Ettorre GM, Santoro E (2007). Clinicopathological features and prognosis of gastric cancer in young European adults. Br J Surg.

[7] Park JC (2009). Clinicopathological aspects and prognostic value with respect to age: an analysis of 3362 consecutive gastric cancer patients. J Surg Oncol.

[8] Al-Refaie WB, Hu CY, Piesters PW, Chang GJ (2011). Gastric adenocarcinoma in young patients: a population-based appraisal. Ann Surg Oncol.

[9] Rona KA (2017). Gastric cancer in the young: an advanced disease with poor prognostic features. J Surg Oncol.

[10] Braga-Neto MB (2018). Clinical characteristics of distal gastric cancer in young adults from Northeastern Brazil. BMC Cancer.

[11] Takatsu Y (2016). Clinicopathological features of gastric cancer in young patients. Gastric Cancer.

[12] Nakamura T, Yao T, Niho Y, Tsuneyoshi M (1999). A clinicopathological study in young patients with gastric carcinoma. J Surg Oncol.

[13] Isobe T (2013). Characteristics and prognosis of gastric cancer in young patients. Oncol Rep.

[14] Tavares A, Gandra A, Viveiros F, Cidade C, Macie J (2013). Analysis of clinicopathologic characteristics and prognosis of gastric cancer in young and older patients. Pathol Oncol Res.

[15] Hsieh FJ, Wang YC, Hsu JT, Liu KH, Yeh CN (2012). Clinicopathological features and prognostic factors of gastric cancer patients aged 40 years or younger. J Surg Oncol.

[16] Wang Z (2016). Clinicopathologic characteristics and prognostic of gastric cancer in young patients. Scand J Gastroenterol.

[17] Zhang J (2018). The prognostic value of age in non-metastatic gastric cancer after gastrectomy: a retrospective study in the U. S. and China. J Cancer.

[18] Japanese Gastric Cancer Association. Japanese gastric cancer treatment guidelines 2014 (ver. 4). *Gastric Cancer* 20, 1–19 (2017). 10.1007/s10120-016-0622-4 PMC5215069 27342689

[19] Xiao H, Liu W, Qu H, Ouyang Y (2018). Peri-operative blood transfusion does not influence overall and disease-free survival after radical gastrectomy for stage II/III gastric cancer: a propensity score matching analysis. J Gastrointest Surg.

[20] Xiao H (2018). Impact of peri-operative blood transfusion on post-operative infections after radical gastrectomy for gastric cancer: a propensity score matching analysis focusing on the timing, amount of transfusion and role of leukocyte depletion. J Cancer Res Clin Oncol.

[21] Cunningham D (2006). Perioperative chemotherapy versus surgery alone for resectable gastroesophageal cancer. N Engl J Med.

[22] Kwon SJ (2011). Evaluation of the 7th UICC TNM Staging System of Gastric Cancer. J Gastric Cance.

[23] Clavien PA (2009). The Clavien-Dindo classification of surgical complications: five-year experience. Ann Surg.

[24] Rubin DB, Thomas N (1996). Matching using estimated propensity scores: relating theory to practice. Biometrics.

[25] Yang T (2016). Perioprioperative blood transfusion does non influence recurrence-free and overall survival after curative resection for hepatocellular carcinoma: a propensity score matching analysis. J Hepatol.

[26] Park HJ (2014). Clinical characteristics and outcomes for gastric cancer patients aged 18–30 years. Gastric Cancer.

[27] Yang Y (2016). The prognostic nutritional index is a predictive indicator of prognosis and postoperative complications in gastric cancer: A meta-analysis. Eur J Surg Oncol.

[28] Tokunaga M (2013). Poor survival rate in patients with post-operative intra-abdominal infectious complications following curative gastrectomy for gastric cancer. Ann Surg Oncol.

[29] Noh SH (2014). Adjuvant capecitabine plus oxaliplatin for gastric cancer after D2 gastrectomy (CLAISSIC): 5-year follow-up of an open-label, randomized phase 3 trial. Lancet Oncol.

[30] Lonjon G (2014). Comparison of treatment effect estimates from prospective nonrandomized studies with propensity score analysis and randomized controlled trials of surgical procedures. Ann Surg.

[31] Datta J (2016). Implications of lymph node staging on selection of adjuvant therapy for gastric cancer in the United States: a propensity score-matched analysis. Ann Surg.

[32] Kim JH (2008). Incidence and long-term outcome of young patients with gastric carcinoma according to sex: does hormonal status affect prognosis?. Arch Surg.

[33] Honda M (2016). Long-term outcomes of laparoscopic versus open surgery for clinical stage I gastric cancer: the LOC-1 study. Ann Surg.

