# Supplementary material for: Clinicopathological features and prognosis of young gastric cancer patients following radical gastrectomy: a propensity score matching analysis
Source: Sci Rep. 2019 Apr 11;9:5943. doi: 10.1038/s41598-019-42406-4 (PMC6459851; doi:10.1038/s41598-019-42406-4)
Supplement: Supplementary file 1 — Supplementary Table 1 and Table 2 [file 41598_2019_42406_MOESM1_ESM.pdf]

Clinicopathological features and prognosis of young gastric cancer patients following radical gastrectomy: a propensity score matching analysis

Wu Liu, M. D., Hu Quan, M. D., Xiaoyan Chen, Yongzhong Ouyang, Hua Xiao, M. D.

Supplementary Table 1. Univariate and multivariate analyses of prognostic factors for overall survival after radical resection of gastric cancer in the entire cohort (n = 1661)

Variables	N	3-, 5-year OS rates	UV *P* value	MV HR (95% CI)	MV *P* value
Gender			0.893		
Male	1095	71.1%, 61.4%			
Female	566	70.2%, 62.0%			
Age (years)			0.724		0.882
> 45	1344	68.9%, 60.9%			
≤ 45	317	70.4%, 64.3%			
Body mass index (kg/m^2)			0.018		0.057
≥ 25	226	77.8%, 66.4%			
< 25	1435	69.7%, 60.9%			
Any comorbidities			0.103		
Yes	493	72.9%, 65.0%			
No	1168	69.9%, 60.4%			
Pre-operative hemoglobin (g/L)			<0.001		0.863
≥ 100	1324	72.9%, 64.3%			
< 100	337	61.8%, 51.1%			
Pre-operative albumin (g/L)			0.046		0.583
≥ 35	1277	71.8%, 63.4%			
< 35	384	67.1%, 57.0%			
Surgical procedure			<0.001		0.320
Laproscopy	198	78.9%, 73.2%			
Open	1463	68.7%, 59.9%			
Type of resection			<0.001		<0.001
Total gastrectomy	375	46.3%, 38.2%		1.811 (1.502-2.184)	
Sub-total gastrectomy	1286	77.1%, 67.5%			
Operation time (min)			<0.001		0.464
≥ 240	383	61.8%, 54.6%			
< 240	1278	72.9%, 62.7%			
Intra-operative blood loss (mL)			<0.001		0.015
≥ 300	335	58.9%, 44.5%		1.286 (1.050-1.575)	
< 300	1326	73.8%, 64.9%			
Tumor location			<0.001		0.793
Lower third	1121	77.6%, 68.3%			
Upper, middle third or diffused	540	56.4%, 47.8%			
Differentiation			<0.001		0.017
Well-differentiated	299	83.8%, 73.6%		1.406 (1.062-1.861)	
Moderate- or poor-differentiated	1362	67.5%, 59.3%			

Tumor size (cm)			<0.001		0.233
≥ 5	594	56.7%, 45.9%			
< 5	1067	78.0%, 70.6%			
Depth of invasion			<0.001		<0.001
T4	1002	58.1%, 49.3%		3.050 (2.309-4.029)	
T1-3	659	91.4%, 85.6%			
Lymph node metastasis			<0.001		<0.001
Yes	991	57.4%, 49.1%		2.772 (2.141-3.587)	
No	670	91.0%, 83.1%			
Peri-operative blood transfusion			<0.001		0.011
Yes	325	57.7%, 48.6%		1.299 (1.061-1.591)	
No	1336	73.9%, 64.9%			

OS, overall survival; CI, confidence interval; HR, hazard ratio; UV, univariate analysis; MV, multivariate analysis.

Supplementary Table 2. Univariate and multivariate analyses of prognostic factors for disease-free survival after radical resection of gastric cancer in the entire cohort (n = 1661)

Variables	N	4-, 5-year DFS rates	UV *P* value	MV HR (95% CI)	MV *P* value
Gender			0.841		
Male	1095	70.1%, 59.3%			
Female	566	69.0%, 61.1%			
Age (years)			0.661		0.483
> 45	1344	67.3%, 59.6%			
≤ 45	317	68.9%, 63.5%			
Body mass index (kg/m^2)			0.037		0.149
≥ 25	226	76.2%, 65.2%			
< 25	1435	68.4%, 59.7%			
Any comorbidities			0.126		
Yes	493	71.6%, 64.5%			
No	1168	69.0%, 57.9%			
Pre-operative hemoglobin (g/L)			<0.001		0.626
≥ 100	1324	72.1%, 63.0%			
< 100	337	60.8%, 49.4%			
Pre-operative albumin (g/L)			0.051		
≥ 35	1277	70.9%, 62.2%			
< 35	384	66.2%, 55.4%			
Surgical procedure			<0.001		0.118
Laproscopy	198	77.1%, 71.8%			
Open	1463	67.3%, 58.6%			
Type of resection			<0.001		<0.001
Total gastrectomy	375	45.4%, 36.5%		1.838 (1.522-2.220)	
Sub-total gastrectomy	1286	76.2%, 66.4%			
Operation time (min)			<0.001		0.102
≥ 240	383	60.7%, 53.2%			
< 240	1278	71.8%, 61.5%			
Intra-operative blood loss (mL)			<0.001		0.080
≥ 300	335	57.8%, 42.6%			
< 300	1326	72.6%, 63.7%			
Tumor location			<0.001		0.399
Lower third	1121	76.4%, 67.2%			
Upper, middle third or diffused	540	55.8%, 45.9%			
Differentiation			<0.001		0.027
Well-differentiated	299	82.8%, 72.5%		1.376 (1.037-1.825)	
Moderate- or poor-differentiated	1362	66.4%, 58.4%			
Tumor size (cm)			<0.001		0.077

≥ 5	594	55.8%, 44.6%			
< 5	1067	76.6%, 69.5%			
Depth of invasion			<0.001		<0.001
T4	1002	57.4%, 47.3%		2.958 (2.238-3.910)	
T1-3	659	89.7%, 84.2%			
Lymph node metastasis			<0.001		<0.001
Yes	991	56.3%, 47.6%		3.13 (2.392-4.095)	
No	670	89.8%, 81.9%			
Peri-operative blood transfusion			<0.001		0.050
Yes	325	54.7%, 44.9%		1.230 (1.000-1.511)	
No	1336	72.4%, 63.9%			

DFS, disease-free survival; CI, confidence interval; HR, hazard ratio; UV, univariate analysis; MV, multivariate analysis.
